# Supplementary material for: Editing eIF4E in the Watermelon Genome Using CRISPR/Cas9 Technology Confers Resistance to ZYMV
Source: Int J Mol Sci. 2024 Oct 25;25(21):11468. doi: 10.3390/ijms252111468 (PMC11546804; doi:10.3390/ijms252111468)
Supplement: Supplementary file 1 [file ijms-25-11468-s001.zip › ijms-3204663-supplementary.pdf]

Table S1 The primer sequences used in this study

Primer name	Sequence（5′–3′）	Application
CleIF4E1-qF	CCGACCGATCTATACCTTCTCTACTG	For the qPCR
CleIF4E1-qR	AGTCCATTTCCCTCCATTCGCAC	
CleIF4E2-qF	CCAAGACAGCAACAAATGAGGCTG	For the qPCR
CleIF4E2-qR	CCTTCTCAAGTCCTCATGAAAGCTG	
CleIF4E3-qF	GCTCAACTCTGCTCAACCACTTC	For the qPCR
CleIF4E3-qR	GAAGGCCATCTTTGAGGTCTCGAGC	
CleIFiso4E-qF	ATGGGTGGTCCCGTGTTATTCG	For the qPCR
CleIFiso4E-qR	CCATATTCCGGGGACTTGTCGC	
CleIF4E1-708F	ATGGTAGTCGAAGAGACGATCAAAGC	Amplification the ORF of CleIF4E1
CleIF4E1-708R	TCACACTGAATATTTGTTCTTTGCATGTC	
ZYMV-738F	GAAAGGTCAAAGGAAACAACAGCAC	For the detection of ZYMV
ZYMV-738R	AAGAAGCGTTGCTTTTAAACGGTGGAAAGTCC	
CGMMV-654F	CGTGGTAAGCGGCATTCTAAACCTC	For the detection of CGMMV
CGMMV-654R	CCGCAAACCAATGAGCAAACCG	
